# Effect of Preoperative Vitamin D Deficiency on Hypocalcemia in Patients with Acute Hypoparathyroidism after Thyroidectomy

**DOI:** 10.1155/2020/5162496

**Published:** 2020-07-22

**Authors:** Peeradon Vibhatavata, Pongthep Pisarnturakit, Mongkol Boonsripitayanon, Paveena Pithuksurachai, Nattachet Plengvidhya, Sirinart Sirinvaravong

**Affiliations:** ^1^Division of Endocrinology and Metabolism, Department of Medicine, Faculty of Medicine Siriraj Hospital, Mahidol University, Bangkok, Thailand; ^2^Division of Head and Neck Surgery, Department of Surgery, Faculty of Medicine Siriraj Hospital, Mahidol University, Bangkok, Thailand; ^3^Department of Otorhinolaryngology, Faculty of Medicine Siriraj Hospital, Mahidol University, Bangkok, Thailand

## Abstract

Postoperative hypoparathyroidism is a common complication of total or completion thyroidectomy. The association between preoperative vitamin D deficiency (VDD) and the development of more severe postoperative hypocalcemia is still unclear. *Objectives*. To evaluate the effect of preoperative VDD on severity of hypocalcemia in patients with hypoparathyroidism following thyroidectomy. *Methods*. Patients who developed acute hypoparathyroidism after total or completion thyroidectomy, defined as postoperative parathyroid hormone (PTH) level <15 pg/mL and albumin-adjusted calcium level <8.6 mg/dL, were prospectively recruited. Patients were divided into two groups according to their preoperative vitamin D status (VDD group: 25-hydroxyvitamin D (25(OH)D) level <20 ng/mL; non-VDD group: 25(OH) level ≥20 ng/mL). The primary outcome was severity of hypocalcemia in postoperative hypoparathyroidism. Significant hypocalcemia was defined as calcium level ≤7.5 mg/dL. *Results*. Forty-three patients (21 VDD, 22 non-VDD) were enrolled. Serum total albumin-adjusted calcium level was significantly lower in the VDD group (7.71 ± 0.5 vs. 8.16 ± 0.4 mg/dL, *p* < 0.01), and the incidence of symptomatic hypocalcemia was significantly higher in the VDD group (43% vs. 9%, *p*=0.01). The median maximal daily supplementary dose of elemental calcium was significantly higher in the VDD group (2,400 vs. 1,500 mg/day, *p*=0.02). Length of hospital stay was nonsignificantly longer in the VDD group (*p*=0.06). Preoperative vitamin D level <19.6 ng/mL could predict significant and symptomatic hypocalcemia in postoperative hypoparathyroidism with sensitivity of 90% and 82% and specificity of 70% and 69%, respectively. *Conclusion*. VDD is an independent risk factor for both significant and symptomatic hypocalcemia in hypoparathyroidism patients after thyroid surgery.

## 1. Introduction

Postoperative hypocalcemia is a common complication of total or completion thyroidectomy [1, 2]. The prevalence of this condition was reported to be 10% to 50% and 0.5% to 4.4% for transient and permanent hypocalcemia, respectively [3, 4]. The major cause of hypocalcemia after thyroid surgery is acute hypoparathyroidism resulting from injury, devascularization, or inadvertent removal of the parathyroid glands during thyroidectomy [5, 6]. This complication can cause morbidity, including symptomatic hypocalcemia, requirement for postoperative calcium and vitamin D supplementation, and longer length of hospital stay.

Vitamin D deficiency (VDD), which is usually defined as serum 25-hydroxyvitamin D [25(OH)D] levels less than 20 ng/mL [7], is a global health problem. In Thailand, the prevalence of VDD in adults increased from 31.8% to 43% from 2011 to 2013 [8]. The active form of vitamin D is essential for regulation of calcium homeostasis by increasing intestinal calcium absorption and bone resorption. It also regulates parathyroid hormone (PTH) secretion [9]. PTH is a major regulator of renal 1*α*-hydroxylase (CYP27B1) activity, thereby increasing the level of active metabolites of vitamin D (1,25-dihydroxyvitamin D; calcitriol) in the circulation; its absence, as in hypoparathyroidism, results in low serum calcium and ultimately manifests itself as symptomatic hypocalcemia [10].

Secondary hyperparathyroidism occurs in vitamin D deficiency which is believed to serve as a compensatory mechanism to maintain eucalcemia by enhancing bone resorption and renal calcium reabsorption to offset decreased intestinal calcium absorption from low vitamin D level. Previous studies hypothesized that patients with postoperative hypoparathyroidism whose preoperative vitamin D level was low were at increased risk for lower serum calcium levels and symptomatic hypocalcemia due to the loss of compensatory role of PTH [11, 12]. Many studies were conducted to investigate the association of vitamin D status with postoperative hypocalcemia and yielded mixed results. Some studies concluded that low preoperative serum 25(OH)D level was a risk factor for postoperative hypocalcemia [13–16]. However, other studies have refuted this correlation [17–19]. Both preoperative 25(OH)D level and postoperative PTH level have been reported as independent predictors for postthyroidectomy hypocalcemia. Kim et al. found that preoperative 25(OH)D level <10 ng/mL in subgroup of patients with postoperative PTH level <15 pg/mL after thyroidectomy had 9.6 times higher risk of symptomatic hypocalcemia [15]. Interestingly, one study showed that postoperative PTH level, rather than preoperative 25(OH)D status, was a predictor of postoperative hypocalcemia [19]. Based on these diverse results, we aimed to accurately investigate the role of low 25(OH)D level in developing hypocalcemia by eliminating PTH level as a confounder.

Accordingly, the purpose of this study was to evaluate the effect of preoperative VDD on severity of hypocalcemia in patients with acute hypoparathyroidism, defined as an initial postoperative PTH level <15 pg/mL after total or completion thyroidectomy.

## 2. Materials and Methods

This is a prospective observational study in patients who developed acute hypoparathyroidism following thyroidectomy, defined by intact parathyroid hormone (iPTH) level less than 15 pg/mL with coexisting serum albumin-adjusted calcium level <8.6 mg/dL on postoperative day 0 (POD-0).

We enrolled all consecutive adult patients who were scheduled to undergo total or completion thyroidectomy between August 2018 and March 2019 in the Division of Head and Neck Surgery of the Department of Surgery or the Department of Otorhinolaryngology, Faculty of Medicine Siriraj Hospital, Mahidol University, Bangkok, Thailand. All patients provided written informed consent prior to surgery. The study's protocol was approved by the Siriraj Institutional Review Board (SIRB) (COA no. 330/2561). Patients were excluded if they had preexisting calcemic disorders; had dialysis-dependent end-stage renal disease; underwent gastric bypass surgery prior to enrollment; and/or were on a medication that is known to affect calcium levels, including active or semiactive vitamin D, corticosteroids, thiazide diuretics, and/or antiepileptic agents. Preoperative serum 25(OH)D, calcium, albumin, phosphate, and intact parathyroid hormone (iPTH) levels were measured the day before surgery. After thyroidectomy, serum calcium, phosphate, iPTH, and albumin levels were measured simultaneously on two occasions. The first measurement was taken on postoperative day 0 (POD-0) after the patient arrived at the ward. The second measurement was taken the following morning (POD-1). Serum magnesium levels were measured on POD-0. Magnesium replacement was given if patients developed hypomagnesemia (serum magnesium level <1.6 mg/dL).

Based on the laboratory results of POD-0 and POD-1, only patients who developed postoperative biochemical hypoparathyroidism were included in the study for analysis of correlation between preoperative vitamin D and postoperative hypocalcemia. Study participants were divided into two groups based on their preoperative vitamin D status, measured the day before surgery: VDD group, 25-hydroxyvitamin D (25(OH)D) level <20 ng/mL; non-VDD group, 25(OH) level ≥20 ng/mL. Clinical parameters and postoperative outcomes were prospectively compared between the two groups.

All patients who developed acute biochemical hypoparathyroidism received oral calcium carbonate and/or alfacalcidol (1*α*-hydroxyvitamin D3) supplementation. Intravenous calcium gluconate was administered in symptomatic patients. The initial dosages and further adjustment were determined according to the discretion of the attending physicians. Serum calcium, iPTH, phosphate, and albumin levels were measured the next morning (POD-2), and oral calcium and/or alfacalcidol dosages were modified according to serum calcium levels. Remeasurement of serum calcium, albumin, and iPTH levels was performed at our outpatient clinic 1 to 2 weeks after hospital discharge.

### 2.1. Laboratory Measurements

Serum 25(OH)D and iPTH levels were measured using an electrochemiluminescence immunoassay (ECLIA) using a cobas^®^ 8000 modular analyzer (Hitachi High-Technologies Corporation, Tokyo, Japan), and serum total calcium, phosphate, albumin, and magnesium levels were measured by standard methods using a cobas^®^ 8000 modular analyzer (Hitachi High-Technologies Corporation) in the Department of Clinical Pathology, Faculty of Medicine Siriraj Hospital, Mahidol University, Bangkok, Thailand. The reference ranges of laboratory parameters were as follows: 8.8–10.2 mg/dL for serum calcium; 2.5–4.5 mg/dL for serum phosphate; ≥30–100 ng/mL for serum 25(OH)D; and 10–65 pg/mL for serum iPTH. The intra-assay and interassay coefficients of variation (CVs) of these tests were 0.7% and 0.8% for serum calcium; 0.8% and 1.4% for serum phosphate; 3.3% and 4.5% for serum 25(OH)D; and 0.9% and 1.5% for serum iPTH, respectively.

### 2.2. Definitions

Vitamin D deficiency was defined as a serum 25(OH)D level <20 ng/mL based on the current Endocrine Society consensus guideline [8]. Biochemical hypocalcemia was defined as an albumin-adjusted calcium level <8.6 mg/dL (albumin-adjusted calcium = [0.8 × (4-serum albumin) + serum total calcium]). Biochemical hypoparathyroidism was defined as an iPTH level below the lower limit of the laboratory standard (15 pg/mL) coexisting with hypocalcemia. Significant hypocalcemia was defined as an albumin-adjusted calcium level ≤7.5 mg/dL. Symptomatic hypocalcemia was defined as an albumin-adjusted calcium level <8.6 mg/dL in addition to the presence of any symptoms and signs of hypocalcemia, such as numbness and paresthesia of the fingertips, toes, and perioral area; presence of Chvostek's sign and/or Trousseau's sign; or tetany [18].

### 2.3. Outcome Measurement

The primary outcome was the severity of hypocalcemia in postoperative hypoparathyroidism during hospitalization. Secondary outcomes included the incidence of symptomatic hypocalcemia, dose of calcium and vitamin D supplementation to maintain normal serum calcium level during admission, length of hospital stay, and determination of preoperative serum 25(OH)D level that significantly increased the risk of significant and symptomatic hypocalcemia.

### 2.4. Statistical Analysis

The statistical analysis was performed using IBM SPSS ver. 20.0 (SPSS, Inc., Chicago, IL, USA). The number of patients required in each group to provide a power of 80% for detecting a difference in the primary outcome was twenty-one. Data are presented as mean ± standard deviation (SD) or median and interquartile range (IQR) for continuous variables, and as absolute numbers and percentages for categorical variables. Statistical differences between groups were assessed using Student's *t*-test or Mann–Whitney *U* test for continuous variables, depending on distribution. Chi-square test or Fisher's exact test was performed to compare categorical variables. Odds ratio with 95% confidence interval was used to describe the results of univariate and multivariate analyses to investigate the effect of evaluated factors on significant hypocalcemia and symptomatic hypocalcemia. A *p* value < 0.05 was considered to be statistically significant.

## 3. Results

Eighty-five patients underwent total or completion thyroidectomy during the study period. Of those, 43 patients (50.6%) developed postoperative biochemical hypoparathyroidism. Twenty-one patients had a preoperative serum 25(OH)D level of <20 ng/mL, whereas 22 patients had a level ≥20 ng/mL (*p*=0.23). The baseline demographic and clinical characteristics of patients in both groups are shown in Table 1. The mean preoperative 25(OH)D levels in VDD and non-VDD group were 15.44 and 26.89 ng/mL, respectively. The ranges were 9.3–19.9 ng/mL in VDD group and 21.1–35.6 ng/mL in non-VDD group. The majority of patients in both groups were female (86% vs. 91%, *p*=0.66), and the mean age of patients in the VDD group was lower than that in the non-VDD group (46 ± 17 vs. 55 ± 12 years, *p*=0.07). There were no significant differences between two groups for type of thyroidectomy (*p*=1.00), type of thyroid cancer (*p*=0.42), neck dissection procedure (*p*=0.16), operative time (*p*=0.26), or weight of excised thyroid gland (*p*=0.84). Two patients (9.5%) in the VDD group and 2 patients (9.1%) in the non-VDD group received ergocalciferol for treatment of VDD preoperatively (*p*=1.00).

As shown in Table 2, the number of intraoperative parathyroid glands identified and preserved was not different between groups, and so did the histopathological report of parathyroid tissue.

The pre-and postoperative clinical and biochemical parameters of both groups are shown in Table 3. As compared to the non-VDD group, the mean preoperative serum 25(OH)D level was significantly lower in the VDD group (15.44 vs. 26.89 ng/mL, *p* < 0.01), and the mean preoperative serum iPTH level was nonsignificantly higher in the VDD group (52.86 vs. 47.45 pg/mL, *p*=0.34). No difference was found between the VDD and non-VDD groups relative to preoperative albumin-adjusted calcium (9.14 vs. 9.26 mg/dL, *p*=0.32), preoperative serum phosphate (3.45 vs. 3.45 mg/dL, *p*=0.99), or preoperative estimated glomerular filtration rate (eGFR) (105.1 vs. 95.6 ml/min, *p*=0.26). There was no difference in postoperative iPTH levels between VDD and non-VDD patients on POD-0 (11.40 vs. 9.66 pg/mL, *p*=0.11), POD-1 (11.58 vs. 9.54 pg/mL, *p*=0.05), or POD-2 (12.78 vs. 9.67 pg/mL, *p*=0.13). There was also no difference observed between groups in postoperative phosphate or magnesium levels. Postoperative serum albumin-adjusted calcium levels were significantly lower in the VDD group than in the non-VDD group on POD-0 (8.12 vs. 8.42 mg/dL, *p*=0.01), POD-1 (8.09 vs. 8.37 mg/dL, *p*=0.09), and POD-2 (7.83 vs. 8.52 mg/dL, *p* < 0.01). The lowest serum albumin-adjusted calcium level during admission was significantly lower in the VDD group than in the non-VDD group (7.71 vs. 8.16 mg/dL, *p* < 0.01). The data showed a similar percentage of iPTH decline between preoperation and POD-0 in both groups (75.79% in the VDD group vs. 77.96% in the non-VDD group, *p*=0.58). The rate of symptomatic hypocalcemia was significantly higher in the VDD group than in the non-VDD group (43% vs. 9%, *p*=0.01). There was a trend towards a higher rate of intravenous calcium treatment for hypocalcemia in the VDD group (33% vs. 9%, *p*=0.07). The median maximal supplementary dose of elemental calcium per day was significantly higher in the VDD group than in the non-VDD group (2,400 vs. 1,500 mg/day, *p*=0.02), while the median maximal supplementary dose of alfacalcidol per day was similar between groups (*p*=0.08). Length of hospital stay was nonsignificantly longer in the VDD group (*p*=0.06). None of the patients in our cohort required readmission due to hypocalcemia.

**Figure 1:**
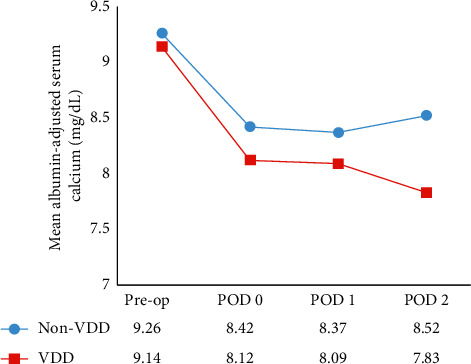
Mean albumin-adjusted serum calcium level.

**Figure 2:**
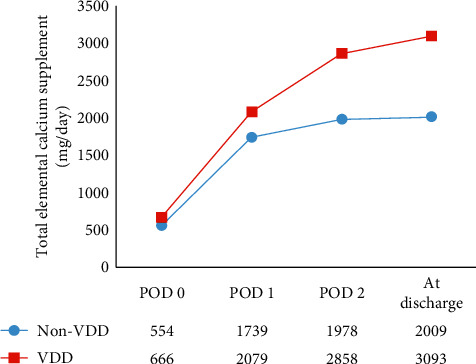
Elemental calcium supplementation per day.

The postoperative albumin-adjusted calcium and the dosage of calcium supplementation in the VDD and non-VDD groups are shown in Figures 1 and 2, respectively.

The serum albumin-adjusted calcium level which was measured at the first outpatient visit, 1–2 weeks following hospital discharge, was not different between VDD group and non-VDD group (9.07 vs. 8.92 mg/dL, *p*=0.59). Intact PTH level, obtained during the outpatient visit, was also similar in both groups (19.59 pg/mL in VDD group vs. 16.30 pg/mL in non-VDD group, *p*=0.42).

One year after thyroidectomy, 71.4% (15 of 21) of patients in VDD group vs. 68.2% (15 of 22) of patients in non-VDD group were able to discontinue alfacalcidol (*p*=0.82). However, duration of independence from alfacalcidol was significantly shorter in the non-VDD group than the VDD group (16 days vs. 50 days from POD-0, *p*=0.001). There was no difference in duration of independence from calcium supplement between both groups (51 days vs. 37 days from POD-0, *p*=0.24) (Table 4).

Subgroup analysis was performed, comparing postoperative outcomes between patients with preoperative 25(OH)D level <12 ng/mL (*n* = 5) and ≥20 ng/mL (*n* = 22). We observed a trend of higher rate of symptomatic hypocalcemia (40% vs. 9%, *p*=0.14); intravenous calcium requirement for treatment of hypocalcemia (40% vs. 9%, *p*=0.14); and higher median maximal supplementary dose of elemental calcium per day (2,600 vs. 1,500 mg/day, *p*=0.11) in the former group. However, the difference did not reach statistical significance. The median maximal dose of alfacalcidol (1 vs. 1 mcg/day, *p*=0.08) and length of hospital stay (6 vs. 4 days, *p*=0.59) were similar between these 2 subgroups.

In both univariate and multivariate analyses, preoperative 25(OH)D level was found to be significantly associated with postoperative significant hypocalcemia (Table 5) and symptomatic hypocalcemia (Table 6).

**Figure 3:**
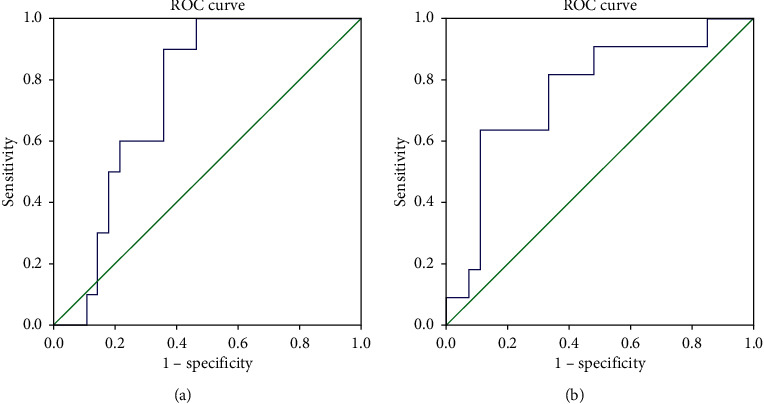
Receiver operating curve analysis to determine the 25(OH)D cut-off level for predicting significant. (a) Significant hypocalcemia: sensitivity 90%, specificity 70%, area under the curve = 0.785. (b) Symptomatic hypocalcemia: sensitivity 82%, specificity 69%, area under the curve = 0.784.

Receiver operating curve analysis revealed that preoperative 25(OH)D level of 19.6 ng/mL could be considered a reliable cut-off value for predicting significant postoperative hypocalcemia (AUC: 0.79, 95% CI: 0.7–0.9; *p*=0.007), with sensitivity, specificity, positive predictive value, and negative predictive value of 90%, 70%, 47%, and 96%, respectively. The same 19.6 ng/mL preoperative 25(OH)D level cut-off was also shown to be effective in predicting postoperative symptomatic hypocalcemia (AUC: 0.78, 95% CI: 0.6–0.9; *p*=0.005) with sensitivity, specificity, positive predictive value, and negative predictive value of 82%, 69%, 47%, and 92%, respectively (Figure 3).

## 4. Discussion

Vitamin D is a fat-soluble vitamin that is derived from cholesterol. It is metabolized in the liver to 25(OH)D, which is used to determine a person's vitamin D status, and then it is converted by 1*α*-hydroxylase to its active form, 1,25-dihydroxyvitamin D [1,25(OH)_2_D], in the kidneys [20–22]. The enzyme 1*α*-hydroxylase is stimulated by elevation of parathyroid hormone level, as well as low serum calcium or phosphorus levels, and it is inhibited by serum fibroblast growth factor-23 (FGF-23) [23]. 1,25(OH)_2_D acts on the small bowel to increase intestinal calcium and phosphate absorption. It also increases bone resorption [24]. VDD state leads to a compensatory increase in PTH secretion to maintain serum calcium concentrations [25].

VDD, which is defined as a 25(OH)D level <20 mg/mL, is a common health problem worldwide [26]. The prevalence of VDD in Thailand was estimated to range from 31.8% to 43% in the general population, and those living in an urban area were found to be at increased risk for developing VDD [7]. We hypothesized that patients with preoperative VDD who developed acute hypoparathyroidism after thyroid surgery would develop more severe hypocalcemia.

Predictors of postthyroidectomy hypocalcemia have been explored in several studies. Preoperative vitamin D status is one of the parameters that are widely studied; however, the results are heterogeneous. No correlation of preoperative 25(OH)D level and development of postoperative hypocalcemia was reported by some of those studies. Lin et al. reported that vitamin D deficiency, using a preoperative 25(OH)D threshold of either <30 ng/mL or <20 ng/mL, was not significantly associated with increased risk of postoperative hypocalcemia, but all of the patients in that study were routinely prescribed calcium and cholecalciferol postoperatively [18]. Griffin et al. reported that 25(OH)D levels had no effect on the risk of postthyroidectomy hypocalcemia. The mean vitamin D levels in patients with and without postoperative hypocalcemia were 15.6 and 15.8 mg/dL, respectively. The mean PTH level was significantly lower in patients who developed hypocalcemia than in patients who did not [11]. Lang et al. found that patients with 25(OH)D levels <10 ng/mL had significantly lower postoperative serum calcium levels, but greater PTH response to hypocalcemia [27]. They concluded that preoperative measurement of 25(OH)D levels appeared unnecessary due to a lack of correlation between 25(OH)D levels and postoperative calcium kinetics. In contrast, Tripathi et al. conducted a prospective study on 35 patients who underwent thyroid surgery, showing positive correlation between preoperative VDD and serum calcium levels in the early postoperative period [16]. Al-Khatib et al. reported that postoperative biochemical and symptomatic hypocalcemia were significantly associated with low levels of 25(OH)D [13]. Alkhalili et al. showed that preoperative VDD was associated with an increased risk of postoperative hypocalcemia and a prolonged length of hospital stay in patients who underwent total thyroidectomy [14].

The reasons for these conflicting results could be due to differences in the biochemical cut-off levels used to define vitamin D deficiency and biochemical hypocalcemia [19], as well as the type of thyroid surgery (with or without neck dissection). Moreover, time interval between 25(OH)D level measurement and surgery was substantially different among studies. Alkhalili et al. [14] reported the mean interval of 137 days as opposed to that within 1 month in another study [15]. Moreover, time interval was not clearly defined in several other studies [11, 13, 18]. Furthermore, the majority of these studies were of retrospective nature, and the critical data such as preoperative vitamin D levels were missing which may limit the significance of the research findings.

Some studies showed that low postoperative PTH level was predictive of postthyroidectomy hypocalcemia, as an independent predictor or in combination with low preoperative vitamin D. Soares et al. prospectively evaluated 47 patients who were undergoing total thyroidectomy and reported that postoperative hypocalcemia was significantly associated with low postoperative PTH level [19]. Kim et al. demonstrated that low vitamin D level in conjunction with postoperative PTH <15 pg/mL increased risk of symptomatic hypocalcemia [15].

The purpose of our study was to accurately investigate the effect of low vitamin D level in developing hypocalcemia, and postoperative PTH could serve as a confounder; therefore, we decided to study only patients with postoperative PTH level <15 pg/mL. We measured 25(OH)D level the day before surgery to ensure its reflection on the true vitamin D status. Our results showed that overall (at nadir and at each time point) postoperative serum albumin-adjusted calcium in the VDD group was lower than in the non-VDD group. The VDD group also needed higher dosage of calcium supplement. Even though all patients were given alfacalcidol supplement (in order to bypass renal 1*α*-hydroxylase in patients with hypoparathyroidism) with a median dose of 1 microgram per day, an adequate storage pool of vitamin D was essential to prevent significant and symptomatic hypocalcemia. In addition, there was an insignificant trend of higher rate of symptomatic hypocalcemia and intravenous calcium requirement in subgroup of patients with preoperative 25(OH)D level <12 ng/mL compared to those with level >20 ng/mL. These findings were probably due to a small number of patients who had 25(OH)D level less than 12 ng/mL (*n* = 5).

The mechanism through which vitamin D deficiency might result in increased postthyroidectomy hypocalcemia is largely speculative [14]. Serum calcium levels are regulated by multiple factors including parathyroid hormone and vitamin D levels. Vitamin D-deficient patients absorb less calcium from the gut, resulting in compensatory increase in PTH level to maintain eucalcemia by PTH-mediated bone and renal calcium reabsorption. In postoperative hypoparathyroidism, the injured parathyroid glands are unable to increase the secretion of PTH and, thus, predispose VDD patients to more severe hypocalcemia because their calcium regulation is more dependent on serum PTH levels than that of patients without vitamin D insufficiency [13, 15, 16, 28].

To our knowledge, this is the first prospective study on patients with acute hypoparathyroidism after total or completion thyroidectomy that was designed to investigate the relationship between VDD and postoperative significant and symptomatic hypocalcemia. Similar prospective studies were conducted by Thipathi et al. and Soares et al.; however, both studies included all patients undergoing thyroid surgery, regardless of postoperative PTH levels [19]. A study by Chia et al. was also prospective in nature, but in contrast to other studies, they used 1,25(OH)_2_D level to represent preoperative vitamin D status [17].

We also identified 19.6 ng/mL as an appropriate and reliable 25(OH)D cut-off value for predicting significant and symptomatic hypocalcemia with high sensitivity and high negative predictive value. Patients were found to be less likely to develop significant and symptomatic hypocalcemia if the preoperative 25(OH)D level was above this cut-off level. Kim et al. [15] and Kirkby-Bott et al. [28] reported that a preoperative 25(OH)D level greater than 20 ng/mL reduced the risk of symptomatic hypocalcemia, which is similar to the results of our study.

The limitations of our study include a relatively small number of patients and the fact that all included patients were enrolled from a single center. Moreover, there was a lack of treatment protocol for hypocalcemia, and the dosage adjustment of calcium and vitamin D supplement was determined according to the discretion of the attending physician.

In conclusion, we demonstrated that preoperative vitamin D deficiency is an important risk factor for significant and symptomatic hypocalcemia in postoperative hypoparathyroidism after thyroid surgery. Patients with vitamin D deficiency require higher dosage of calcium supplement to maintain normocalcemia. Normal storage pool of vitamin D is essential in patients who are particularly prone to postoperative hypoparathyroidism (for example, for those undergoing total thyroidectomy and/or neck dissection for thyroid malignancy, prior to central neck surgery, or in centers with low-volume thyroid surgeons) to avoid hypocalcemia after thyroid surgery. Serum 25(OH)D levels above 20 ng/mL reduced the risk of postoperative significant and symptomatic hypocalcemia in these patients.

## Figures and Tables

**Table 1:** Baseline demographic and clinical characteristics of studied patients.

Characteristics	VDD (*n* = 21)	Non-VDD (*n* = 22)	*p* value
Demographics			
Female sex, *n* (%)	18 (85.7%)	20 (90.9%)	0.66
Age (years), mean ± SD	46.3 ± 17	55 ± 12	0.07
Malignant thyroid disease, *n* (%)	14 (66.7%)	12 (54.5%)	0.42
Papillary, *n*	13	10	
Follicular, *n*	0	1	
Medullary, *n*	1	1	
Benign thyroid disease, *n* (%)	7 (33.3%)	10 (45.5%)	0.42
Graves' disease, *n*	2	5	
Multinodular toxic goiter, *n*	0	1	
Multinodular euthyroid goiter, *n*	5	4	
Total thyroidectomy, *n* (%)	19 (90.5%)	20 (90.9%)	1.00
Neck dissection, *n* (%)			
Not performed	14 (66.7%)	19 (86.4%)	0.353
CCND	2 (9.1%)	1 (4.5%)	
CCND + MRND	5 (23.8%)	2 (9.1%)	
Duration of operation (min), median (IQR)	190 (105–225)	118 (90–205)	0.26
Weight of excised thyroid gland (g), median (IQR)	39 (26–150)	56 (25–129)	0.84

A *p* value < 0.05 indicates statistical significance. VDD: vitamin D deficiency; SD: standard deviation; CCND: central compartment neck dissection; MRND: modified radical neck dissection; IQR: interquartile range.

**Table 2:** Comparison of parathyroid glands preservation between VDD and non-VDD groups.

Preservation status	VDD (*n* = 21)	Non-VDD (*n* = 22)	*p* value
Parathyroid glands identified and preserved during surgery, *n* (%)			
1 parathyroid gland	2 (9.5%)	0	0.686
2 parathyroid glands	6 (28.6%)	8 (36.4%)	
3 parathyroid glands	3 (14.3%)	3 (13.6%)	
4 parathyroid glands	10 (47.6%)	11 (50.0%)	

Parathyroid glands identified on histological examination, *n* (%)			
0 parathyroid glands	13 (61.9%)	16 (72.7%)	0.415
1 parathyroid gland	8 (38.1%)	5 (22.7%)	
2 parathyroid glands	0	1 (4.5%)	

A *p* value < 0.05 indicates statistical significance. VDD: vitamin D deficiency.

**Table 3:** Comparison of clinical and biochemical parameters during hospitalization between VDD and non-VDD groups.

Parameters	VDD (*n* = 21)	Non-VDD (*n* = 22)	*p* value
Preoperative laboratory values			
25(OH)D level (ng/mL)	15.44 ± 3.3	26.89 ± 4.2	<0.01
PTH (pg/mL)	52.86 ± 20.8	47.45 ± 16.1	0.34
Albumin-adjusted calcium (mg/dL)	9.14 ± 0.4	9.26 ± 0.4	0.32
Phosphate (mg/dL)	3.45 ± 0.6	3.45 ± 0.4	0.99
eGFR (ml/min/1.73 m^2^)	105.07 ± 22.4	95.59 ± 20.3	0.26
Postoperative laboratory values			
PTH (pg/mL)			
Postoperative day 0	11.40 ± 3.59	9.66 ± 3.29	0.11
Postoperative day 1	11.58 ± 3.26	9.54 ± 3.37	0.05
Postoperative day 2	12.78 ± 7.82	9.67 ± 3.63	0.13
Albumin-adjusted calcium (mg/dL)			
Postoperative day 0	8.12 ± 0.45	8.42 ± 0.23	0.01
Postoperative day 1	8.09 ± 0.43	8.34 ± 0.54	0.09
Postoperative day 2	7.83 ± 0.61	8.52 ± 0.60	<0.01
Lowest albumin-adjusted calcium (mg/dL)	7.71 ± 0.50	8.16 ± 0.35	<0.01
Phosphate (mg/dL)			
Postoperative day 0	3.82 ± 0.78	3.93 ± 0.64	0.64
Postoperative day 1	4.42 ± 0.86	4.47 ± 0.95	0.85
Postoperative day 2	4.27 ± 0.91	4.26 ± 1.25	0.99
Magnesium POD-0 (mg/dL)	1.65 ± 0.18	1.74 ± 0.12	0.05
Percentage of PTH decline (%)	75.79 ± 13.4	77.96 ± 12.4	0.58
Symptomatic hypocalcemia	9 (42.9%)	2 (9.1%)	0.01
Required intravenous calcium treatment	7 (33.3%)	2 (9.1%)	0.07
Maximal calcium supplement per day (mg)	2400 (1800, 3600)	1500 (1200, 2400)	0.02
Maximal alfacalcidol supplement per day (mcg)	1 (1, 2)	1 (0.5, 1)	0.08
Length of hospital stay (days)	5 (4, 8)	4 (4, 6)	0.06
Patients requiring readmission	0 (0.0%)	0 (0.0%)	—

Data are presented as mean ± SD, number and percentage, or median and IQR. A *p* value < 0.05 indicates statistical significance. VDD: vitamin D deficiency; 25(OH)D: 25-hydroxyvitamin D; PTH: parathyroid hormone; eGFR: estimated glomerular filtration rate; POD: postoperative day; SD: standard deviation; IQR: interquartile range.

**Table 4:** Clinical and biochemical parameters at follow-up visits after hospital discharge.

Parameters	VDD (*n* = 21)	Non-VDD (*n* = 22)	*p* value
First follow-up visit			
PTH (pg/mL)	19.59 ± 17.22	16.30 ± 7.88	0.42
Albumin-adjusted calcium (mg/dL)	9.07 ± 1.05	8.92 ± 0.77	0.59

Independence from alfacalcidol supplement^*∗*^			
Independence from alfacalcidol supplement, *n* (%)	15 (71.43%)	15 (68.18%)	0.82
Time to independence from alfacalcidol (days) (median, IQR)	50 (36, 61)	16 (8, 30)	0.001

Data are presented as mean ± SD, number and percentage, or median and IQR. A *p* value < 0.05 indicates statistical significance. VDD: vitamin D deficiency; PTH: parathyroid hormone; SD: standard deviation; IQR: interquartile range. ^*∗*^Data were collected at 1 year after total thyroidectomy.

**Table 5:** Univariate and multivariate analysis for factors associated with significant postoperative hypocalcemia.

Covariates	Univariate analysis			Multivariate analysis		
	*β*-Coefficient	Odds ratio (95% CI)	*p* value	*β*-Coefficient	Odds ratio (95% CI)	*p* value
Preoperative 25(OH)D level (ng/mL)	−0.18	0.83 (0.72–0.96)	0.01	−0.37	0.69 (0.52–0.90)	0.007
Duration of operation (minutes)	0.002	1.00 (0.99–1.01)	0.26			
Intraoperative preservation of parathyroid glands	−0.22	0.80 (0.40–1.62)	0.54			
Weight of excised gland (g)	−0.01	0.99 (0.98–1.01)	0.42			
PTH level on postoperative day 0 (pg/mL)	−0.10	0.91 (0.73–1.12)	0.37			
Percentage of PTH decline (%)	0.06	1.06 (0.98–1.14)	0.17			
Postoperative magnesium level (mg/dL)	−4.77	0.01 (0.00–1.12)	0.06			

A *p* value < 0.05 indicates statistical significance. CI: confidence interval; 25(OH)D: 25-hydroxyvitamin D; PTH: parathyroid hormone; g: gram.

**Table 6:** Univariate and multivariate analysis for factors associated with symptomatic hypocalcemia.

Covariates	Univariate analysis			Multivariate analysis		
	*β*-Coefficient	Odds ratio (95% CI)	*p* value	*β*-Coefficient	Odds ratio (95% CI)	*p* value
Preoperative 25(OH)D level (ng/mL)	−0.18	0.84 (0.73–0.96)	0.01	−0.21	0.81 (0.68–0.97)	0.02
Duration of operation (minute)	0.01	1.00 (1.00–1.01)	0.07			
Intraoperative preservation of parathyroid glands	0.15	1.17 (0.58–2.35)	0.67			
Weight of excised gland (g)	0.01	1.01 (1.00–1.01)	0.29			
PTH level on postoperative day 0 (pg/mL)	−0.01	1.00 (0.81–1.21)	0.91			
Percentage of PTH decline (%)	0.04	1.04 (0.97–1.11)	0.26			
Postoperative magnesium level (mg/dL)	−3.34	0.04 (0.00–3.33)	0.15			

A *p* value < 0.05 indicates statistical significance. CI: confidence interval; 25(OH)D: 25-hydroxyvitamin D; PTH: parathyroid hormone; g: gram.

## Data Availability

The data used to support the findings of this study are available from the corresponding author upon reasonable request.
